# Combinatorial Effects of Aromatic 1,3-Disubstituted Ureas and Fluoride on *In vitro* Inhibition of *Streptococcus mutans* Biofilm Formation

**DOI:** 10.3389/fmicb.2016.00861

**Published:** 2016-06-06

**Authors:** Gurmeet Kaur, P. Balamurugan, C. Uma Maheswari, A. Anitha, S. Adline Princy

**Affiliations:** ^1^Quorum Sensing Laboratory, Centre for Research in Infectious Diseases, School of Chemical and Biotechnology, SASTRA UniversityThanjavur, India; ^2^Organic Synthesis Group, Department of Chemistry, School of Chemical and Biotechnology, SASTRA UniversityThanjavur, India

**Keywords:** *Streptococcus mutans*, dental caries, fluoride resistant, quorum sensing, synergism, multidrug resistant

## Abstract

Dental caries occur as a result of disequilibrium between acid producing pathogenic bacteria and alkali generating commensal bacteria within a dental biofilm (dental plaque). *Streptococcus mutans* has been reported as a primary cariogenic pathogen associated with dental caries. Emergence of multidrug resistant as well as fluoride resistant strains of *S. mutans* due to over use of various antibiotics are a rising problem and prompted the researchers worldwide to search for alternative therapies. In this perspective, the present study was aimed to screen selective inhibitors against ComA, a bacteriocin associated ABC transporter, involved in the quorum sensing of *S. mutans*. In light of our present *in silico* findings, 1,3-disubstituted urea derivatives which had better affinity to ComA were chemically synthesized in the present study for *in vitro* evaluation of *S. mutans* biofilm inhibition. The results revealed that 1,3-disubstituted urea derivatives showed good biofilm inhibition. In addition, synthesized compounds exhibited potent synergy with a very low concentration of fluoride (31.25–62.5 ppm) in inhibiting the biofilm formation of *S. mutans* without affecting the bacterial growth. Further, the results were supported by confocal laser scanning microscopy. On the whole, from our experimental results we conclude that the combinatorial application of fluoride and disubstituted ureas has a potential synergistic effect which has a promising approach in combating multidrug resistant and fluoride resistant *S. mutans* in dental caries management.

## Introduction

*Streptococcus mutans* has been reported as a primary cariogenic bacterial pathogen in causing dental caries, along with other acidogenic bacteria such as *Lactobacillus casei*, Actinomyces (Kaur et al., 2015). In addition, this bacterium has also been reported to cause chronic infective endocarditis which may cause significant morbidity and mortality (Persson, 2012). *S. mutans* has established various characteristic mechanisms for its unhindered growth and survival in the complex microbial community of the oral cavity. Major virulence factors such as acidogenicity, aciduricity, and the ability to synthesize extracellular polysaccharides from carbohydrates such as sucrose using enzymes such as glucosyltransferases (GTFs) and collagenanses, reported to have ability to bind and degrade collagen type I, a major component of dentin (Han et al., 2006) are a few among such mechanisms (Hasan et al., 2012). The glucan synthesis plays a crucial role in biofilm formation of dentine commonly known as dental plaque (Lynch et al., 2013). Recent advances in understanding the communication between bacterial communities have established that like various other bacteria, *S. mutans* also regulate the process of biofilm formation through quorum sensing (QS) system (Miller and Bassler, 2001). QS circuit in *S. mutans* consists of two component signal transduction system (TCSTS) that specifically detect and respond to the signaling peptide known as Competence Stimulating Peptide (CSP) (Kleerebezem et al., 1997). The CSP is synthesized as a propeptide by *comC* which is then processed and matured by an ABC transporter ComA with the help of an accessory protein ComB and finally secreted to the extracellular environment. The secreted peptide is detected by the ComD, a histidine kinase protein receptor resulting in phosphorylation of its cytoplasmic response regulator, ComR thus, enabling the cell to respond to the peptide via regulation of gene expression controlling various virulence factors such as genetic competence and biofilm formation (Kotake et al., 2008).

Biofilm formation is one of the significant characteristics in various infectious diseases as it provides various advantages to bacteria such as protection from host defense system, sequestration of nutrients, utilization of community benefits, and protection from various antimicrobials since it acts as a diffusion barrier for those agents to penetrate deep into the biofilms (Senadheera and Cvitkovitch, 2008). Targeting one of the crucial components involved in QS circuit can lead to biofilm inhibition (Qi et al., 2005; Rasmussen and Givskov, 2006; Ravichandiran et al., 2013). Development of novel drugs against biofilm formation, a major virulence factor in dental caries aids in the effective clearance of *S. mutans* when used in combination with very low concentrations of antimicrobials thus combating multidrug resistance (Chen et al., 2016).

Fluoride has been used long as an effective anti-caries agent in various commercial formulations and is the mainstay for caries prevention (Zheng et al., 2015). It exerts major effects by reducing enamel demineralization and enhancing remineralization of early caries lesions (Randall et al., 2014). However, fluoride does not provide complete protection in its currently used preparations and moreover, emergence of fluoride resistant *S. mutans* has also doubted the prolonged use of fluoride (Mitsuhata et al., 2014). Although, fluoride at higher concentrations helps in reduction of dental plaque and also inhibits the growth of dental pathogens, prolonged use of high concentrations of fluoride has caused various side effects such as fluorosis and weakened bones (Cavalli et al., 2011; Santos et al., 2013). Thus, if an additional agent could be administered synergistically with fluoride, it may lead to improved cariostatic and disruptive effects with respect to biofilm formation without increasing fluoride's exposure.

In view of this hypothesis, ComA was identified as a key molecule crucial for the initiation of QS system of *Streptococcus*, which belongs to a family of bacteriocin-associated ATP-binding cassette (ABC) transporters. ABC transporters usually consists of three domains, i.e., N-terminal domain possessing the proteolytic peptidase activity, a transmembrane domain involving six membrane-spanning segments, and a C-terminal ATP-binding domain positioned toward the cytoplasmic face of the membrane. Proteolytic activity of peptidase domains of this family is believed to be involved in cleaving their cognate propeptides after the consensus Gly-Gly motif. The presence of a cysteine residue is essential to the sequence for the proteolytic activity of this family of ABC transporters (Kotake et al., 2008). Recently, the crystal structure of peptidase (PEP) domain involved in the QS pathway of *Streptococcus* was reported by Ishii et al. (2010) and its proteolytic activity substrate recognition mechanism was reported by Kotake et al. (2008). The key involvement of ComA in maturation and secretion of CSP made it as a favorable target for QS inhibition of *S. mutans*. Thus, the aims of the present study were (1) to screen selective inhibitors against ComA, using computational tools utilizing small compound library and synthesize the screened ligand along with its derivatives, (2) to evaluate the synthesized compounds for biofilm inhibition potential against *S. mutans*, and (3) to determine the combinatorial biological activity of the synthesized compounds at lower concentrations of fluoride.

## Materials and methods

### Ligand and protein preparation

The energy minimized 3D coordinates were generated for all ligands and ligand file was prepared for docking using Schrödinger LigPrep software (Xiao et al., 2011). An independent ligand dataset library Zinc database was used for this study (Irwin and Shoichet, 2005). Since none of the inhibitors have been reported for PEP-ComA, thus, comparison studies were not conducted with known inhibitors. The X-ray crystal structure of the PEP domain of ComA (PDB ID-3K8U) was retrieved from PDB database and processed for addition of polar hydrogen and Kollmann charges using Protein Preparation Wizard (PrepWizard) in Maestro (Schrödinger Suite) (Bernstein et al., 1978). The prepared protein was treated to be completely rigid for all docking procedures to minimize the excessive computation cost. A grid box encompassing the complete macromolecule was constructed and used for all docking runs in this study (Osguthorpe et al., 2012). The hydrogen bonds were optimized and protein minimization was carried out using the standard protein preparation protocol. The possible options available for protein minimization included hydrogen only or all-atom with a criterion based on the root-mean-square deviation (RMSD) of the heavy atoms relative to their initial location. Water molecules were retained through the H-bond optimization and minimization stages, as without water molecules, the protein could collapse, or the H-bond networks required for ligand binding would be disrupted. Although, prior to docking, all the water molecules (>5 Ả) were removed. The resultant protein structure was used for further docking studies with the prepared ligands.

### Molecular docking studies

All docking calculations between macromolecule and ligand datasets were performed with Glide (**G**rid based **LI**gand**D**ocking with **E**nergetics) program in Schrödinger suite (Friesner et al., 2004). All the datasets were executed in three consecutive steps. Precisely, HTVS mode (**H**igh-**T**hroughput **V**irtual **S**creening), SP mode (**S**tandard **P**recision), followed by XP mode (e**X**tra**P**recision mode) using the default settings. All the prepared ligands were docked with the target protein using the induced fit docking (IFD) protocol (Friesner et al., 2006). Extensive sampling was conducted by SP mode which defines the interaction sites of ligand with the protein molecule. Resultant G-scores were considered as the ranking criteria for the selection of best docked ligands to the target protein.

### *De novo* drug designing and lead optimization

The selected ligands were screened based on their respective glide scores, interaction pattern at the site of interest in the protein and the functional group of the compounds. The selected ligands were further run for clustering using Schrödinger Canvas module (Hartenfeller and Schneider, 2011). The 3D-Pharmacophore and binary fingerprinting studies optimized the ligands by ruling out the ligands with similar structures and their interaction with the protein. Clustering of ligands was followed by optimization of lead molecules with Schrödinger QuikProp for toxicity analysis of the selected ligands (Singh and Konwar, 2012). The parameters in ADMET and TOPKAT (**TO**xicity**P**rediction by **K**omputer **A**ssisted **T**echnology) was used for the final validation of the selected compounds and deemed to be the potential leads.

### Synthesis of aromatic 1,3-disubstituted ureas

**Figure 1:**
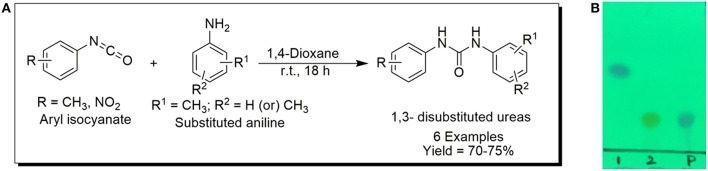
**(A)** Synthesis scheme of 1,3-disubstituted ureas. **(B)** A thin layer chromatogram showing completion of the reaction (30% Ethyl acetate:Hexane). Spot 1 represents reactant 1 (2,4,6-Trimethyl aniline), spot 2 represents (4-nitrophenyl isocyanate), and P represents their corresponding product (1-mesityl-3-(4-nitrophenyl)urea). The difference in the *R*_f_ values and color change of reactants to products indicates the completion of reaction.

The 1,3-disubstituted ureas were synthesized by a simple one pot reaction of aryl isocyanates with the selective amines (Mustafa et al., 2014). The synthesis process is shown as a reaction scheme in Figure 1A. For the synthesis of 1,3-disubstituted ureas, a solution of substituted aniline (3.18 mmol) was mixed in 1,4-dioxane (5 mL) and aryl isocyanate (1.60 mmol) was added drop-wise to the reaction mixture and kept for stirring at room temperature for 18 h. The progress of the reaction was monitored by thin layer chromatography. The difference in the *R*_f_ values of the reactants and products in the reaction mixture indicated the completion of the reaction (Figure 1B). After completion of the reaction, the reaction mixture was cooled down by addition of crushed ice which resulted in formation of products as solids. The solid, thus obtained was filtered and washed several times with cold distilled water followed by washing with hexane to obtain the pure product. The structures of the synthesized products were confirmed by ^1^H and ^13^C NMR spectroscopy.

### Bacterial strains and culture conditions

*S. mutans* MTCC 497 was received from Microbial Type Culture Collection (MTCC), Chandigarh, India and was used as a standard strain in this study. A wild type strain (WT) NG8 was kindly donated by Prof. L. Jeannine Brady, University of Florida, USA. Two clinical isolates of *S. mutans* 4SM (multidrug resistant) and 5SM (fluoride resistant) were received from JSS Medical College, Mysore, India. All the strains were cultured in brain heart infusion broth (BHIB)/agar (HiMedia) at 37°C under stationary condition. When needed, medium was supplemented with 2% sucrose. Sodium fluoride and Chlorhexidine diacetate were purchased from HiMedia (Mumbai, India.)

### Effect of 1, 3-disubstituted ureas on biofilm development in static biofilm model

Ninety six well polystyrene microtiter plates were used for determining minimum biofilm inhibitory concentration (MBIC) and minimum biofilm eradication concentration (MBEC) assay (Kolodkin-Gal et al., 2012; Wang et al., 2015). The assays were carried out against clinical as well as standard isolates of *S. mutans* using two-fold microdilution broth procedure as per the Clinical and Laboratory Standards Institute (CLSI^1^) standards with slight modifications as described below (CLSI Approved Standard—Tenth Edition). *S. mutans* strains were grown overnight in BHIB. The overnight cultures were diluted (1:100) and the initial optical density of the bacterial cultures were adjusted to 0.08 (OD_600_) to achieve 10^8^ CFU/ml approximately. The inoculum was inoculated in fresh medium along with varying concentrations of synthesized compounds ranging from 0.23 to 15 μM as independent experiments (in triplicates). Plates were incubated for 24 h at 37°C without shaking. After incubation, the wells were washed twice with 200 μL of phosphate buffered saline (PBS) gently to remove the non-adherent cells. Adherent cells in the biofilm were fixed by adding 200 μL of 100% methanol prior to staining with 200 μL of 0.2% (w/v) crystal violet (CV) for 20 min. The excess stain was washed twice with PBS and the plates were air dried. The bound CV in the air dried plates were eluted with 200 μL of 33% acetic acid. The biofilm was quantitatively determined by measuring the absorbance at OD_595_ nm in a microtiter plate reader (iMark, BIORAD, Japan). The lowest concentration in which the formation of biofilm is inhibited when compared to the untreated culture control is defined as MBIC i.e., ≥90% (for MBIC_90_ or IC_90_) or ≥50% (for MBIC_50_ or IC_50_).

Similarly, MBEC assay was performed as described elsewhere (Jennings et al., 2014). Briefly, the biofilm of the respective strains were allowed to grow in polystyrene 96 well microtiter plates for 18 h. After incubation, the wells were washed twice with PBS to remove planktonic cells followed by addition of fresh medium along with varying concentrations of synthesized compounds. The plates were incubated for 3 h without shaking followed by CV staining of wells as described earlier. The absorbance at OD_595_ nm was measured. The lowest concentration in which the biofilm was eradicated when compared to the untreated culture control is defined as MBEC i.e., ≥90% (for MBEC_90_ or EC_90_) or ≥50% (for MBEC_50_ or EC_50_). All the assays were performed in triplicates.

### Checkerboard microdilution assay for synergistic studies

Combinatorial effects between synthesized compounds and fluoride as well as chlorhexidine was evaluated by a two dimensional microdilution assay using 96 well microtiter plate (Zheng et al., 2015). The final concentrations of fluoride ranged from 3.90 to 250 ppm and that of chlorhexidine ranged from 0.003125 to 0.2%. The concentration of fluoride was selected where it was not inhibiting bacterial growth and also was not acting as biofilm inhibiting agent. For chlorhexidine, 0.2% is the highest concentrations which is used in commercial formulations. For each combination, each synthesized compound was placed in horizontal rows while fluoride and chlorhexidine were placed into the vertical columns. Data were collected using microtiter plate reader (iMark, BIORAD, Japan) as turbidometric measurements of absorbance (OD_595_) after incubation for 24 h. The synergistic activity was assessed by the currently prevailing two most widely used approaches i.e., using fractional inhibitory concentration index (FICI) and Bliss independence model (ΔE model).

#### Synergy interpretation using FICI

To determine the effect of the combinatorial treatment to be synergistic, indifferent or antagonistic, FICI (a nonparametric method) was calculated (Subramaniam et al., 2014). The following formulas were used to calculate the FICI of a combination.

FICI = FIC of compound A + FIC of compound B

Where,

FIC of compound A = (MIC of compound A in combination)/(MIC of compound A alone).

FIC of compound B = (MIC of compound B in combination)/(MIC of compound B alone).

Synergy was defined as an FIC index value of ≤ 0.5. Indifference or absence of interaction was defined as FICI value >0.5 but < 4. Antagonism was defined as an FICI of >4.

#### Synergy interpretation using bliss independence model

The bliss independence model or BI theory (Goldoni and Johansson, 2007; Sun et al., 2008) is described by the following equation.

(1) $${\text{I}}_{\text{i}}\;=\;({\text{I}}_{\text{A}}\;+\;{\text{I}}_{\text{B}})\;-\;({\text{I}}_{\text{A}}\text{ X }{\text{I}}_{\text{B}})$$

Where,

Ii = predicted percentage of inhibition of the theoretical combination of drug A and B.

I_A_ = experimental percentages of inhibition of drug A.

I_B_ = experimental percentages of inhibition of drug B.

(2) I = 1−E

Where,

E = percentage of growth.

Substituting Equation (2) into (1), a resultant equation is given as

$${\text{E}}_{\text{i}}\;=\;{\text{E}}_{\text{A}}\text{ X }{\text{E}}_{\text{B}}$$

Where,

E_i_ = predicted growth percentage of the theoretical combination of drug A and B.

E_A_ = observed percentage of growth of drug A.

E_B_ = observed percentage of growth of drug B.

Interaction is described by the difference (ΔE) between the predicted and the observed growth percentages at various concentrations as described by the following equation:

$$\Delta \text{E }=\;{\text{E}}_{\text{predicted}}\;-\;{\text{E}}_{\text{observed}}.$$

By the nonparametric approach described by Prichard et al. E_A_ and E_B_ were obtained directly from the experimental data. Due to the nature of interaction, testing with microtiter plates, and a two-fold dilution of either drug results in a ΔE for each drug combination. In each of the three independent experiments, the observed growth percentages derived from the experimental data (observed values) were subtracted from the predicted percentages, and then the average difference of three experiments was calculated. When the average difference as well as its 95% confidence interval among the three replicates was positive, statistically significant synergy was claimed; when the difference as well as its 95% confidence interval was negative, significant antagonism was claimed.

### Confocal laser scanning microscopy imaging of biofilms

Confocal imaging was performed on bacterial cells grown in the form of biofilms on the surface of glass slides. The glass slides were placed inside sterile 50 ml centrifuge tubes containing BHIB with or without the compounds at concentrations that showed maximum biofilm inhibition activity in 96 well microtiter plate experiments (Auty et al., 2001). *S. mutans* was inoculated and the tubes were incubated for 8 h. After the incubation time, slides were processed for CLSM imaging. Briefly, the slides were washed twice with 1X PBS to remove planktonic cells followed by staining of attached biofilm with BacLight LIVE/DEAD stain kit (L-7012; Molecular Probes) according to the manufacturer's instructions. Stained slides were imaged using Carl Zeiss confocal microscope with 63X oil immersion objective.

### Statistical analysis

Graph pad prism software (version 6.01) was used for statistical analysis. One way ANOVA and multiple comparisons were carried out. The minimum level of significance was set at *P* ≤ 0.05. All the assays were conducted in triplicates and the results were expressed as mean ± SD.

## Results

### Docking studies, lead optimization, and toxicity analysis

In the present study, docking analysis revealed several hits that favorably occupy the active site of ComA. Each ligand was further evaluated and the compounds were modified to generate the next generation of molecules with improved binding affinity and less toxicity with the active site. For better activity of ComA inhibitors, ligands should optimally interact with at least one of the four crucial amino acid residues Q11, C17, H96, D112. Lipinski's rule of five predicts a high probability of success or failure of drug likeness for various selected molecules. Eighty one drug like ligands were subsequently guided by Lipinski's rule of three and five, pharmacokinetics (ADME) and pharmacodynamics. Rule of five states that the drug likeness for molecules to comply with three or more of the following rules: Molecular mass < 500 dalton, high lipophilicity, < 5 hydrogen bond donors, and < 10 hydrogen bond acceptors.

Selected compounds were further analyzed using the QuikProp module in Schrodinger for toxicity analysis (ADMET). The properties considered for toxicity prediction were QPlogHERG, QPlogBB (Blood-brain barrier), QPlogKhsa (Human serum albumin), and human oral absorption. Among the selected 81 ligands, 7 ligands satisfied the Lipinski's rule of three and five, and the parameters considered under ADMET properties as given in Table 1. Further, ligand number 5 (ComAI, ComA Inhibitor) was selected based on the ease of synthesis with the available resources.

**Table 1:** **Final selected ligands after TOPKAT and ADMET properties as ComA inhibitors**.

**Ligand No**.	**Structure**	**Glide Score**	**Molecular weight**	**Binding site and other characteristic properties**
1	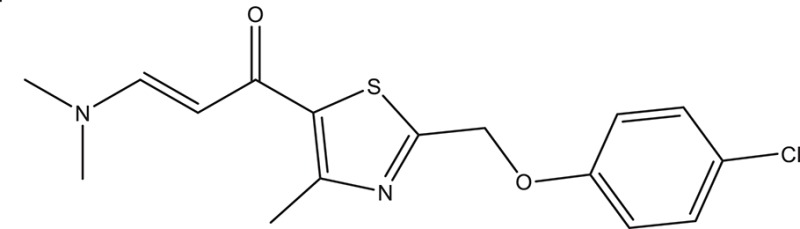	−3.915	336.84	Q11, R93, L94 clogP: 2.780 H-Donors: 0 H-Acceptors: 0 Rotatable bonds-7
2	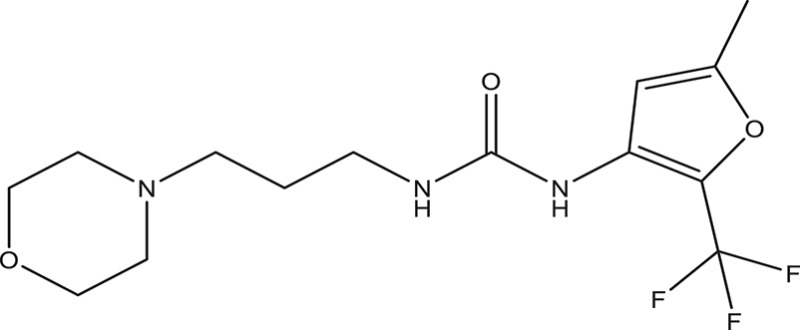	−3.128	335.32	Q11 clogP: 2.510 H-Donors: 2 H-Acceptors: 6 Rotatable bonds: 5
3	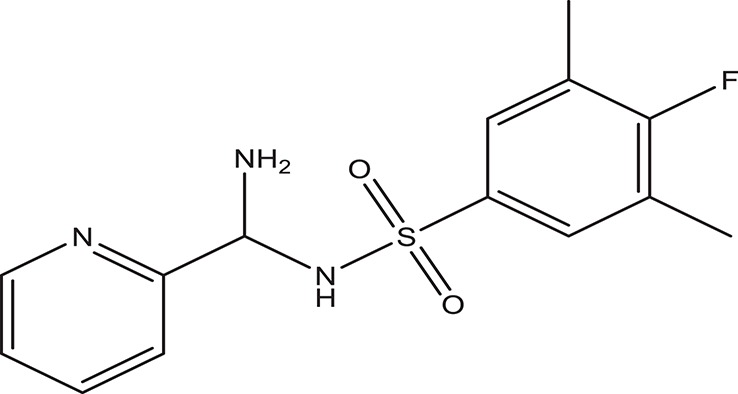	−3.433	307.34	H96, Q95, R93 clogP: 2.220 H-Donors: 2 H-Acceptors: 4 Rotatable bonds: 5
4	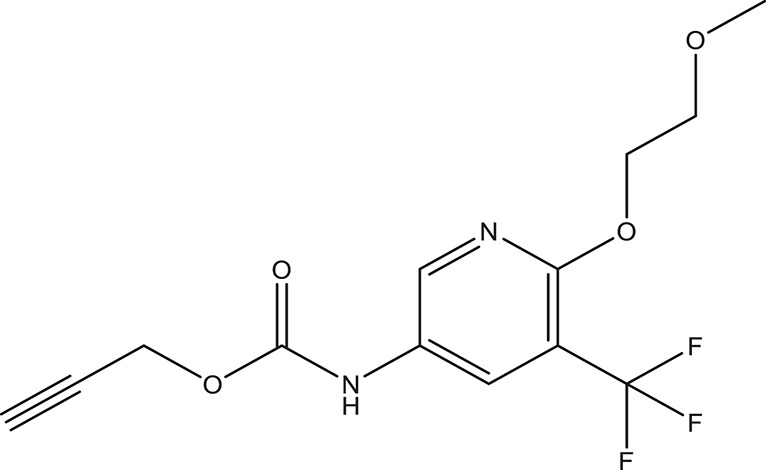	−3.532	318.25	H96, R93, L94 clogP: 2.580 H-Donors: 1 H-Acceptors: 6 Rotatable bonds: 8
5	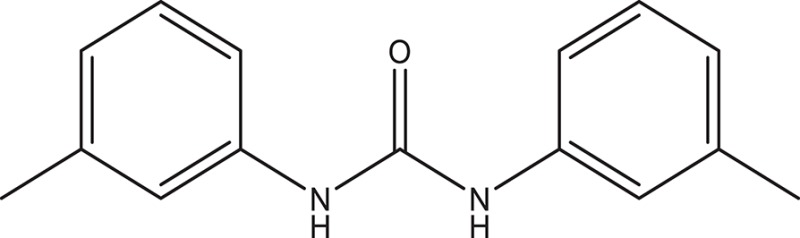	−3.088	240.30	Q11 clogP: 3.350 H-Donors: 2 H-Acceptors: 3 Rotatable bonds: 2
6	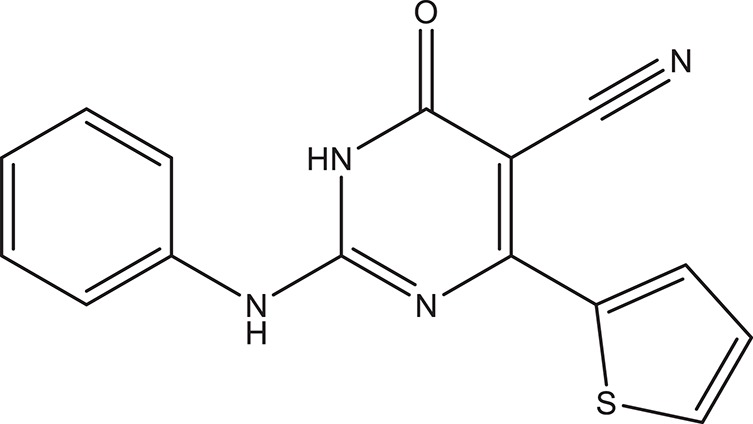	−4.034	294.33	Q11 clogP: 1.830 H-Donors: 2 H-Acceptors: 5 Rotatable bonds: 3
7	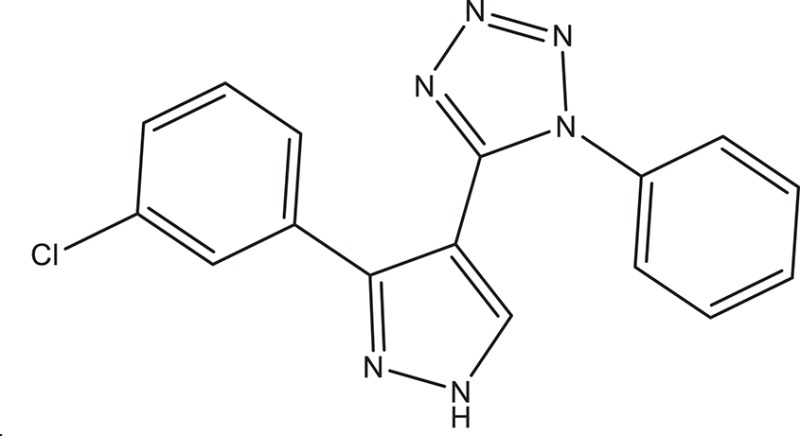	−3.579	322.75	H96, Q95, R93 clogP: 2.860 H-Donors: 1 H-Acceptors: 6 Rotatable bonds: 3

**Figure 2:**
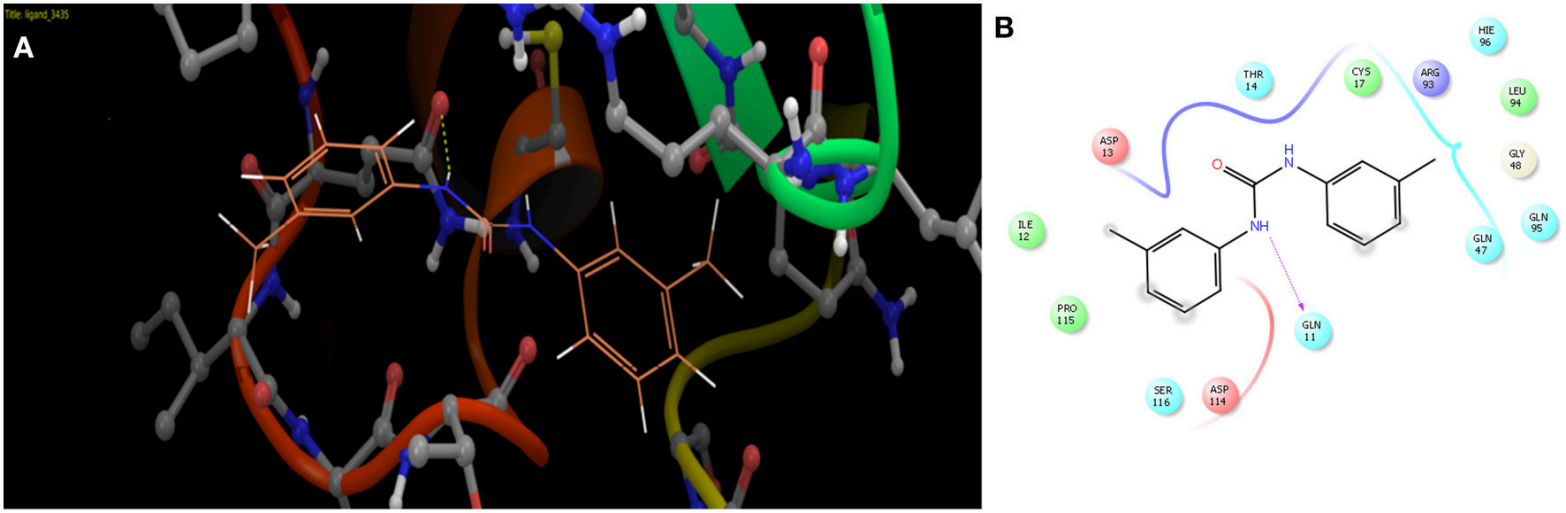
**(A)** Binding of ComAI with PEP domain of ComA through Hydrogen bonding (yellow dotted line). **(B)** Schematic 2D representation of Ligand interaction pattern of ComAI (ComA Inhibitor) with PEP domain of ComA.

**Figure 3:**
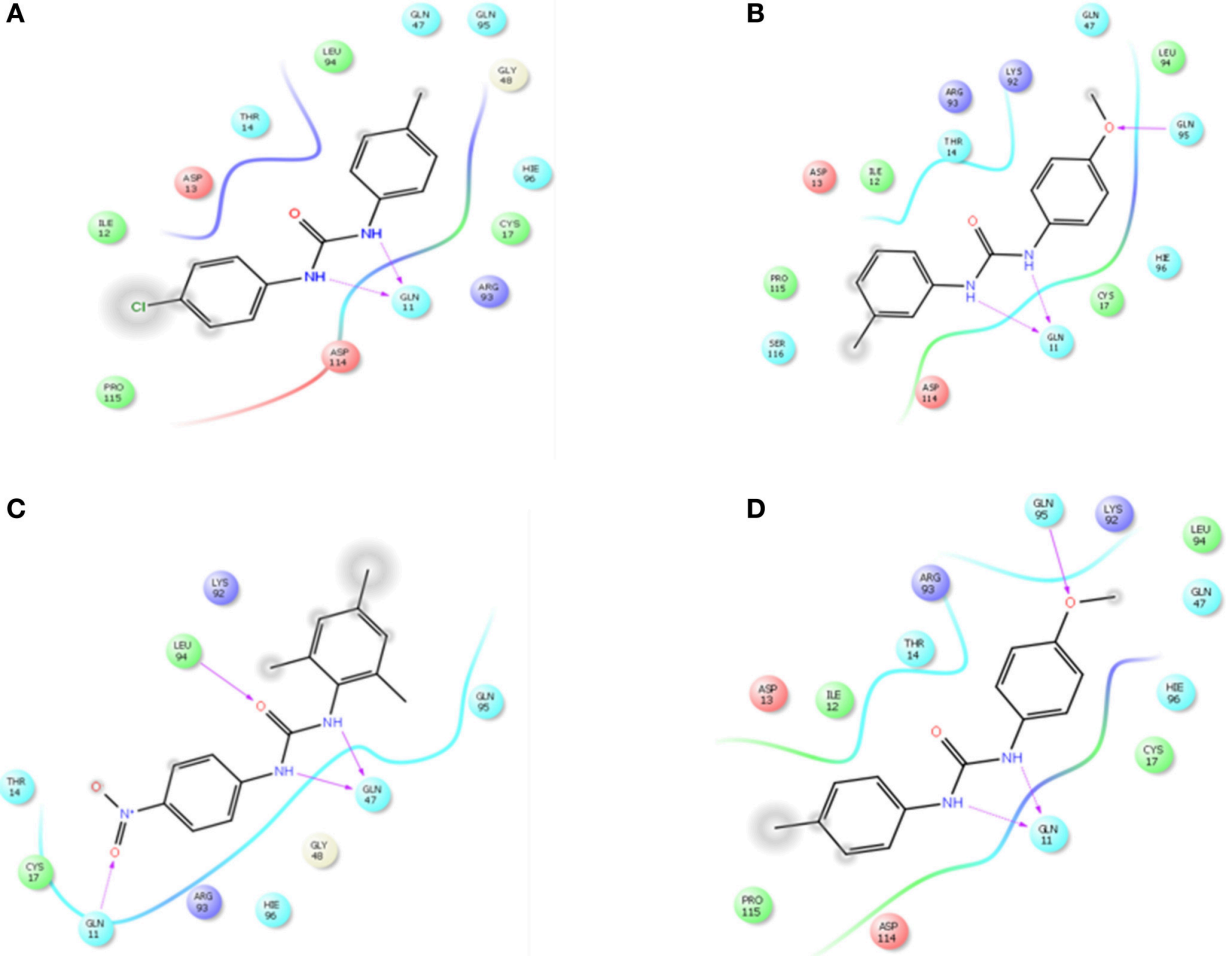
**Schematic 2D representation of interaction of derivatives of ComAI with PEP domain of ComA. (A)** ComAI^1^; **(B)** ComAI^2^; **(C)** ComAI^3^; **(D)** ComAI^4^.

The selected ComAI was further used to generate derivatives with improved binding affinity by substituting various functional groups namely ComAI^1^, ComAI^2^, ComAI^3^, and ComAI^4^. All the derivatives showed better binding affinity to the active site and improved Glide score as compared to the selected ComAI and no toxicity pattern was observed for the derivatives of ComAI as given in Table 2. The ComAI as well as the derived ligands of ComAI interacts and inhibits ComA protein at Q11 through hydrogen bonding along with various other interactions in protein cleft as given in Figures 2A,B, 3A–D. Apart from the selected derivatives, one derivative (ComAI') with no interaction to the active site was also synthesized to compare and validate the computational data with *in vitro* data. Evidently, computational studies using docking tools exhibited excellent outcomes which shows that the selected ligands have potential binding affinity to ComA.

**Table 2:** **Schematic representation of ADMET and TOPKAT properties of ComAI and its derivatives**.

**Name**	**Structure**	**Docking score/interaction site**	**Toxicity prediction(ADMET and TOPKAT)**				
			**Rule of 3 and rule of 5**	**QPlogHERG**	**QPlogBB**	**QPlogKhsa**	**Human oral absorption (%)**
ComAI	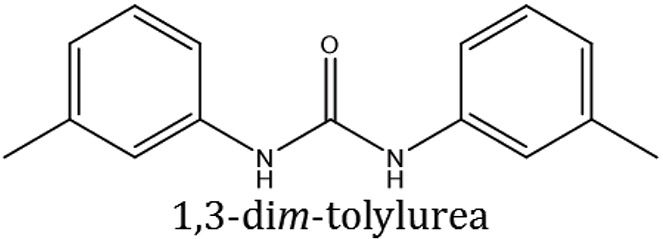	−3.088 Q11	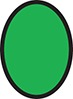	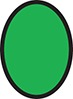	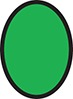	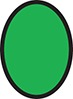	100
Derivatives of ComAI							
ComAI^1^	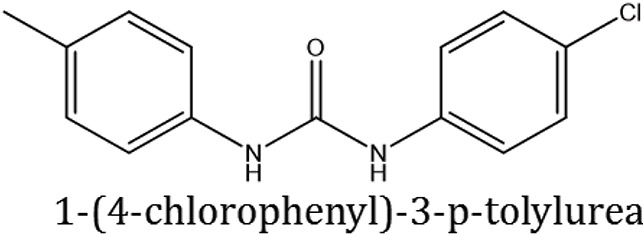	−3.815 Q11	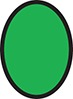	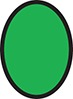	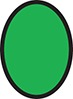	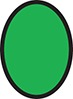	100
ComAI^2^	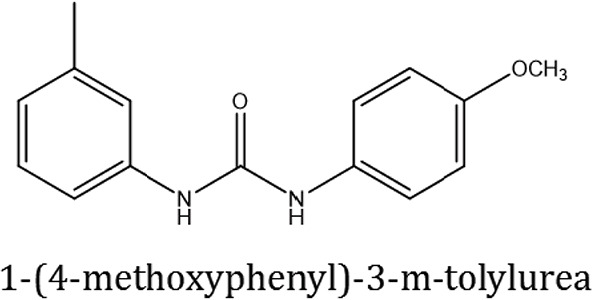	−3.334 Q11,Q95	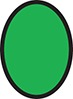	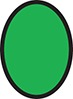	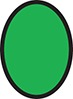	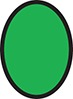	100
ComAI^3^	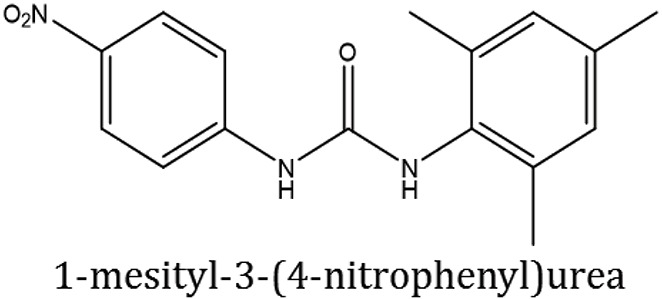	−3.214 Q11, Q47, L94	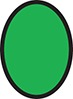	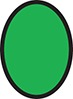	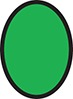	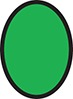	86.7
ComAI^4^	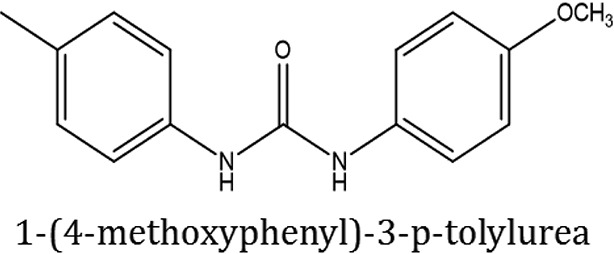	−3.342 Q11,Q95	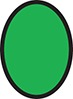	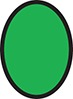	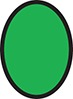	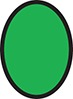	100
ComAI'	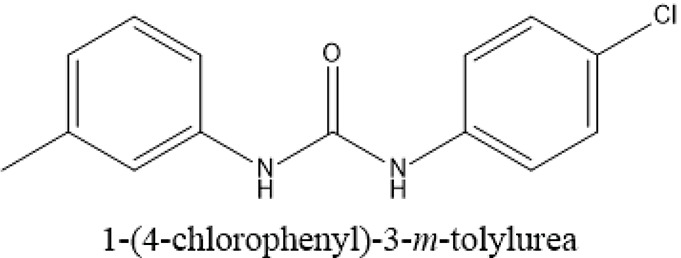	−0.485 –	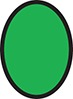	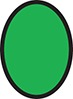	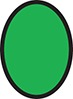	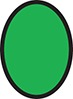	–

*
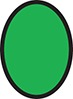
Toxicity free behavior*.

### Synthesis of 1,3-disubstituted ureas

The spectral data (^1^H-NMR and ^13^C-NMR) of all the synthesized compounds were in full agreement with the proposed structures (Table 3). The NMR data is given in Supplementary Material.

**Table 3:** **Structures of reactants and their corresponding products**.

**Ligand name**	**Reactant 1**	**Reactant 2**	**Product**
ComAI	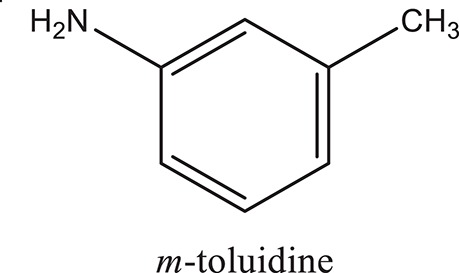	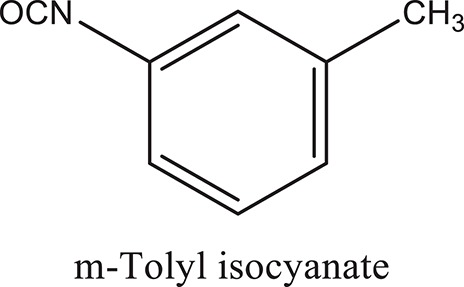	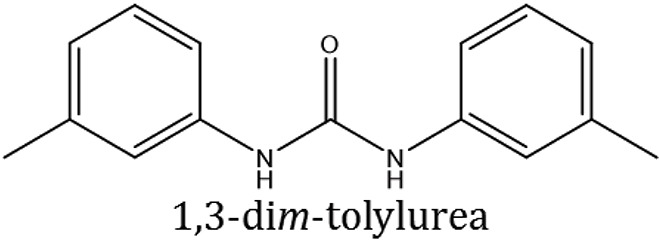
ComAI^1^	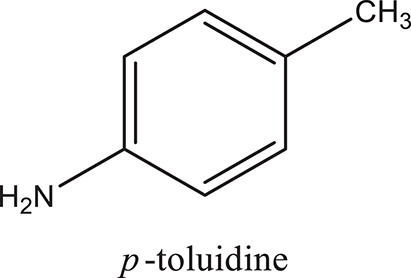	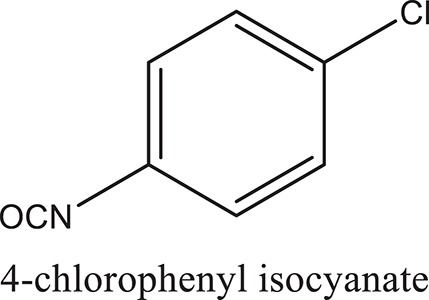	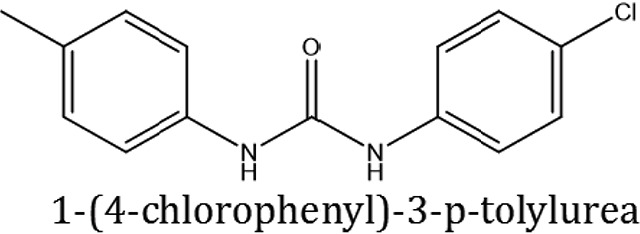
ComAI^2^	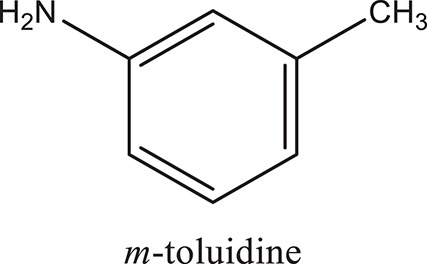	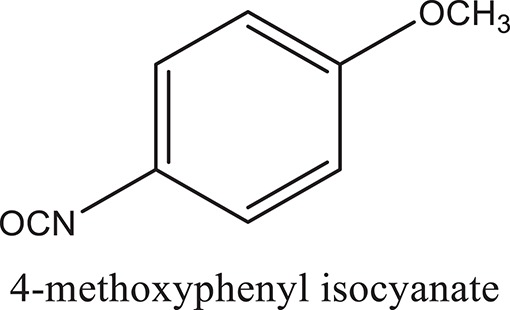	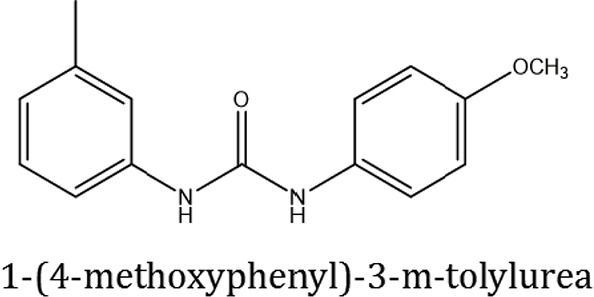
ComAI^3^	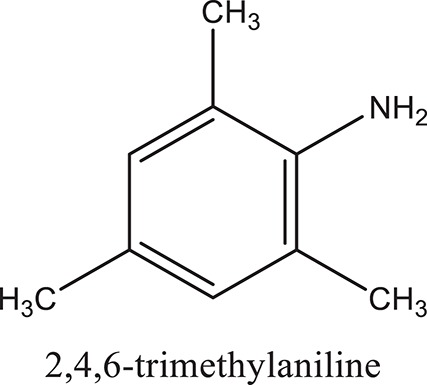	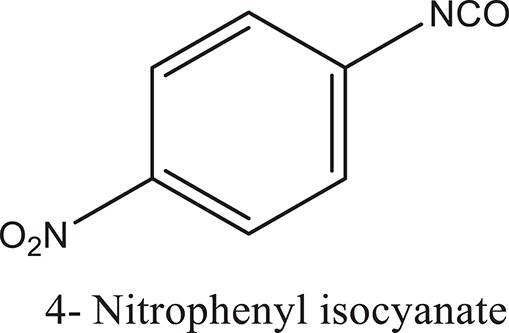	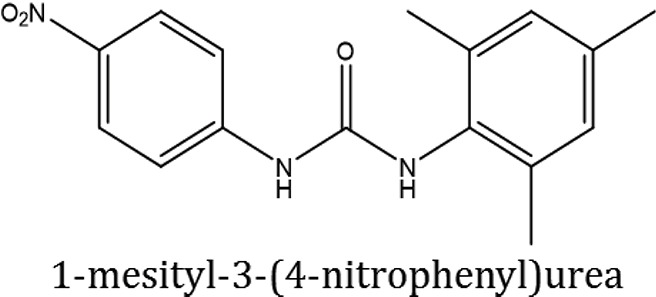
ComAI^4^	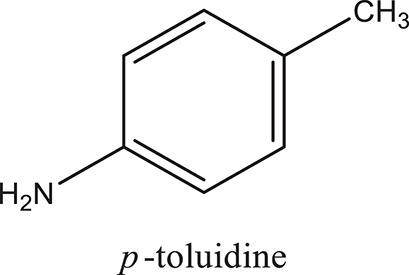	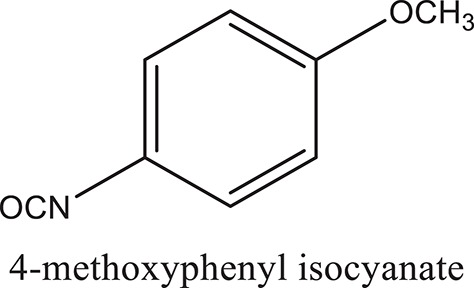	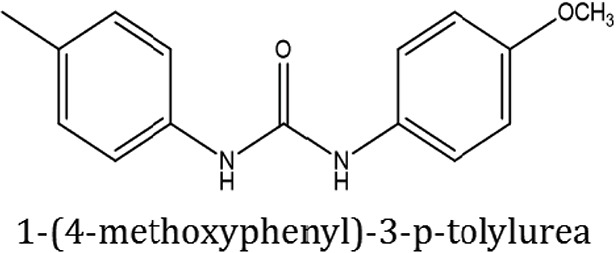
ComAI^1^′	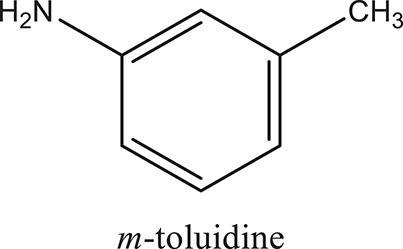	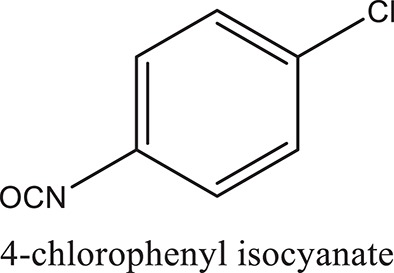	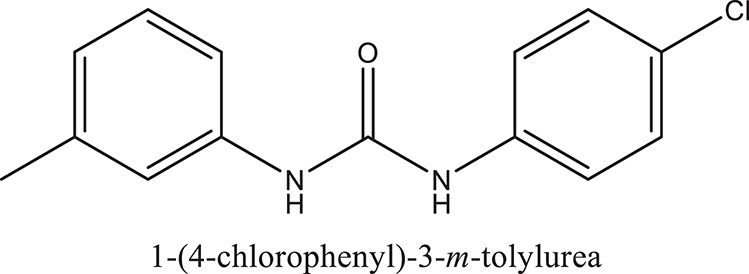

### Anti-biofilm effects of 1, 3-disubstitutedureas on *S. mutans* biofilm development in static biofilm model

Under static conditions, all the compounds except ComAI' were found to have antibiofilm effect. Notably, ComAI and ComAI^1^ showed maximum biofilm inhibition of 50–70% in all the strains with concentrations ranging from 0.23 to 3.75 μM and 0.93 to 1.87 μM respectively. The results were summarized in Table 4. In contrast, no biofilm inhibition was found in 5SM strain by any of the synthesized compounds. In case of eradication assay, eradication of biofilm was not found by any of the compound. Considering better biofilm inhibiting activity of ComAI and ComAI^1^, they were titled as best amongst all the synthesized compounds.

**Table 4:** **IC_50_ and EC_50_ values of ComAI and its derivatives**.

**Compound**	**MTCC497**				**WT**				**4SM**				**5SM**			
	**IC(μM)**		**EC(μM)**		**IC(μM)**		**EC(μM)**		**IC(μM)**		**EC(μM)**		**IC(μM)**		**EC(μM)**	
	**50**	**90**	**50**	**90**	**50**	**90**	**50**	**90**	**50**	**90**	**50**	**90**	**50**	**90**	**50**	**90**
ComAI	3.75	13.5	–	–	14.9	–	–	–	0.39	7.5	–	–	–	14.8	–	–
ComAI^1^	1.89	12.2	–	–	–	–	–	–	0.89	–	–	–	–	12.4	–	–
ComAI^2^	–	13	–	–	11.5	–	–	–	1.78	–	–	–	–	15	–	–
ComAI^3^	4.22	14.9	–	–	–	–	–	–	–	–	–	–	–	13.8	–	–
ComAI^4^	–	–	–	–	0.46	14	–	–	0.41	14.5	–	–	–	14.5	–	–
ComAI^1^′	–	–	–	–	14.7	–	–	–	–	13.5	–	–	–	11.5	–	–

*IC, inhibitory concentration; EC, Eradication Concentration*.

*Strains used: MTCC 497, standard reference strain obtained from MTCC, IMTECH, Chandigarh, India; WT, A wild type strain obtained from kindly donated by Prof. L. Jeannine Brady, University of Florida, USA.; 4SM, Multidrug resistant clinical isolate; 5SM, Fluoride resistant clinical isolate*.

### Drug interactions from synergistic studies

The results of the checkerboard analysis were interpreted using the nonparametric methods based on both the Loewe Additivity (LA) theory and Bliss Independence (BI) theory. In the checkerboard microtiter plate assay, strong synergism was concluded in all the isolates of *S. mutans* analyzed by both the models i.e., FICI and ΔE, and the two models were found to be in correlation for all the tested compounds in the study.

### FICI

The interpretation of the FICI was based on LA theory according to which, a FICI value of ≤ 0.5 revealed synergy, a value of 1–4 revealed indifference, and a value of >4 represented antagonism. ComAI and its derivatives showed synergistic as well as additive effects when used in combination with fluoride as shown in Table 5. In contrast, no synergism was concluded when ComAI and its derivatives were used along with chlorhexidine. Notably, when disubstituted ureas as well as fluoride were used alone, very less or no biofilm inhibition was observed in 5SM strain but considerable biofilm inhibition was observed when used in combination. 1,3-disubstituted ureas were found to inhibit the biofilm in combination at a very low concentration of fluoride i.e., 62.5 ppm. In other strains used in the study, when fluoride was used alone, it showed antibacterial activity at 500 ppm and higher concentrations, but there was no antibiofilm activity at any of the concentrations of fluoride used in the experiment. However, in synergy experiments with 1,3-disubstituted ureas, fluoride showed enhanced biofilm inhibitory effect at concentrations ranging from 31.25 to 62.5 ppm.

**Table 5:** **Synergistic activity of selected ligands with Fluoride (F) and Chlorhexidine (CHX)**.

		**F**	**CHX**	**F**	**CHX**	**F**	**CHX**	**F**	**CHX**	**F**	**CHX**	**F**	**CHX**
MTCC 497	FIC	0.2 (0.05–0.48)	2.8 (1.4–4.0)	0.39 (0.05–0.40)	2.6 (1.6–5.0)	0.05 (0.01–0.17)	3.5 (2.7–5.9)	0.05 (0.02–0.19)	3.8 (2.0–7.1)	–	–	–	–
	I	SYN	NI/ANT	SYN	NI/ANT	SYN	ANT	SYN	ANT	–		–	
WT	FIC	0.01 (0.01–0.05)	0.6 (0.5–2.2)	–	–	0.02 (0.01–0.03)	0.6 (0.5–1.4)	–	–	0.02 (0.02–0.04)	0.6 (0.5–2.4)	0.02 (0.01–0.02)	0.6 (0.5–2.7)
	I	SYN	ADD/NI	–	–	SYN	ADD	–	–	SYN	ADD/NI	SYN	ADD/NI
4SM	FIC	1.1(0.7–1.45)	2.7 (0.6–4.5)	0.52 (0.5–0.6)	2.4 (0.5–4.2)	0.28 (0.28–0.32)	2.2 (0.6–3.9)	–	–	2.41 (2.1–2.5)	3.7 (1.1–6.5)	0.03 (0.03–0.05)	1.5 (1.2–2.7)
	I	ADD	ADD/ANT	ADD	ADD/ANT	ADD	ADD/ANT	–	–	IND	NI/ANT	SYN	NI/ANT
5SM	FIC	0.08 (0.02–0.1)	9.8 (2.0–19.7)	0.2 (0.02–0.1)	10 (2.6–18.5)	0.2 (0.1–0.3)	11.6 (3.7–23.4)	0.08 (0.02–0.1)	11.2 (3.2–25.0)	0.09 (0.02–0.15)	13.7 (6.8–23.5)	0.08 (0.02–0.12)	15.3 (7.8–24.7)
	I	SYN	ANT	SYN	NI/ANT	SYN	ANT	SYN	ANT	SYN	ANT	SYN	ANT

*FIC model -FIC Index of ≤ 0.5 was defined as synergy (SYN), FICI of >4.0 was defined as antagonism (ANT), and indifference [i.e., no interaction (NI) or Additive effect (ADD)] as a FICI of >0.5–4; I corresponds to Interpretation in the above table*.

### Bliss independence model (ΔE model)

The results for BI model were summarized in Table 6. The ΔE value of each tested concentration was the average of triplicate results. To summarize the interaction surface, the sums of the percentages of all statistically significant synergistic (∑SYN) and antagonistic (∑ANT) interactions were calculated. Interactions with < 100% statistically significant interactions were considered as weak, interactions with 100–200% statistically significant interactions were considered as moderate, and interactions with >200% statistically significant interactions were considered as strong, as described previously. In addition, numbers of statistically significant synergistic and antagonistic combinations among all tested drug combinations were calculated for each strain. Bliss independence model was found in coherence with FIC index and confirmed strong synergism among the1,3-disubstituted ureas and fluoride whereas, antagonism as well as indifference was observed in case of chlorhexidine.

**Table 6:** **Delta E model of selected compounds with Fluoride (F) and Chlorhexidine (CHX)**.

**Strains**		**Nonparametric Method**											
		**ComAI**		**ComAI**^**1**^		**ComAI**^**2**^		**ComAI**^**3**^		**ComAI**^**4**^		**ComAI^1^′**	
		**F**	**CHX**	**F**	**CHX**	**F**	**CHX**	**F**	**CHX**	**F**	**CHX**	**F**	**CHX**
MTCC 497	ΔE	1426	−393	1736	−114	1771	−531	1218	−307	–	–	–	–
	I	SS	SA	SS	MA	SS	SA	SS	SA	–		–	
WT	ΔE	1467	44	–	–	1222	22	–	–	1584	84	1730	30
	I	SS	WS	–	–	SS	WS	–	–	SS	WS	SS	WS
4SM	ΔE	98	−329	55	−521	78	−283	–	–	87	−489	1861	−462
	I	WS	SA	WS	SA	WS	SA	–	–	WS	SA	SS	SA
5SM	ΔE	1548	−378	1120	−241	1004	−283	1217	−228	1725	−532	1939	625
	I	SS	SA	SS	SA	SS	SA	SS	SA	SS	SA	SS	SA

*SS, Strong Synergism; SA, Strong Antagonism; WS, Weak Synergism; MA, Moderate Antagonism*.

### Confocal laser scanning microscopy imaging of biofilms

**Figure 4:**
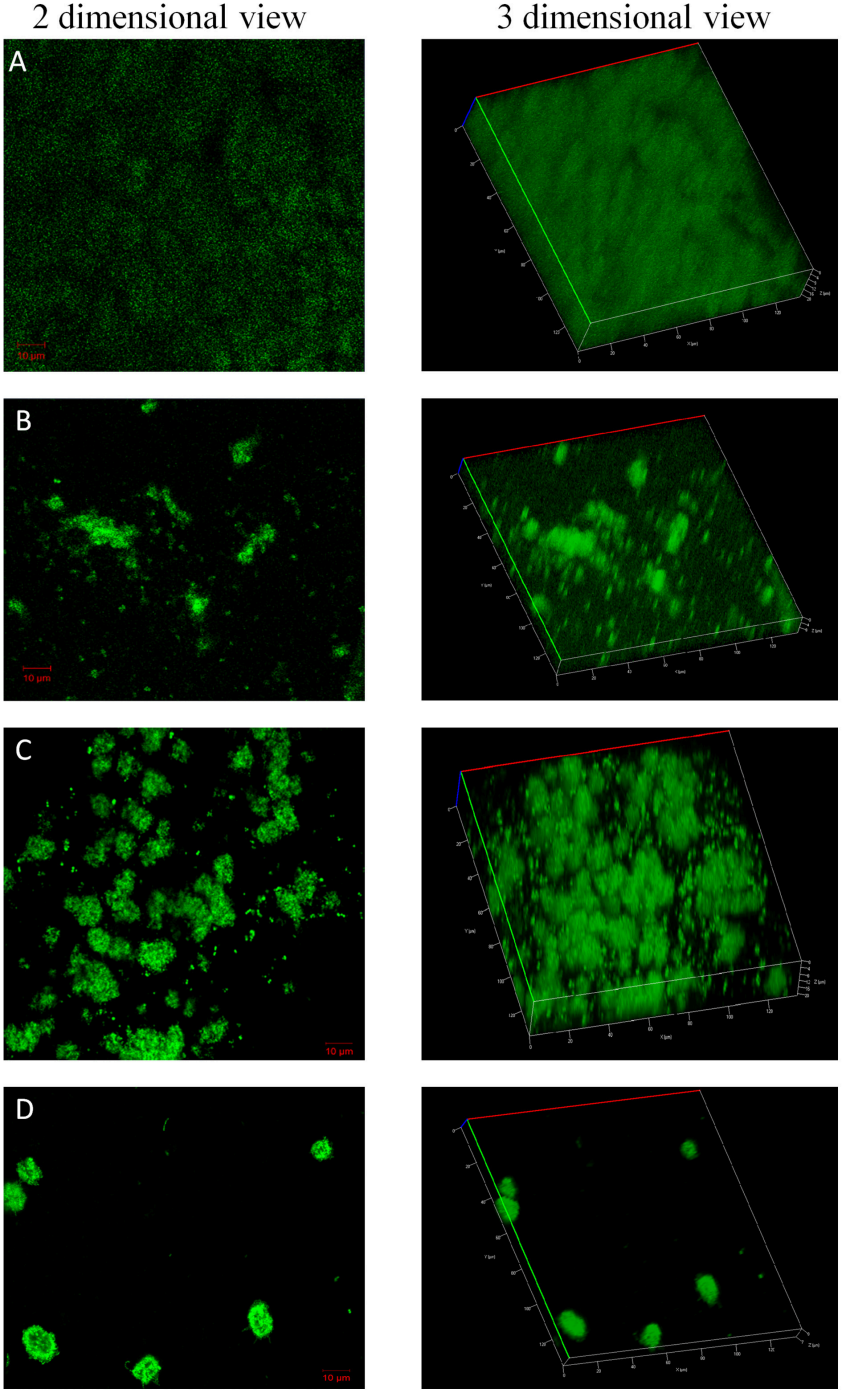
**Effect of ComAI treatments alone as well as in synergy on *S. mutans*MTCC 497 biofilms**. Live/Dead staining of bacterial biofilm observed under confocal laser scanning microscopy (green color-Syto 9). **(A)** Control biofilm, **(B)** ComAI treated biofilm (3.75 μM), **(C)** Fluoride treated biofilm (500 ppm), **(D)** Synergistic activity of ComAI and fluoride (31.25 ppm fluoride and 3.75 μM ComAI).

**Figure 5:**
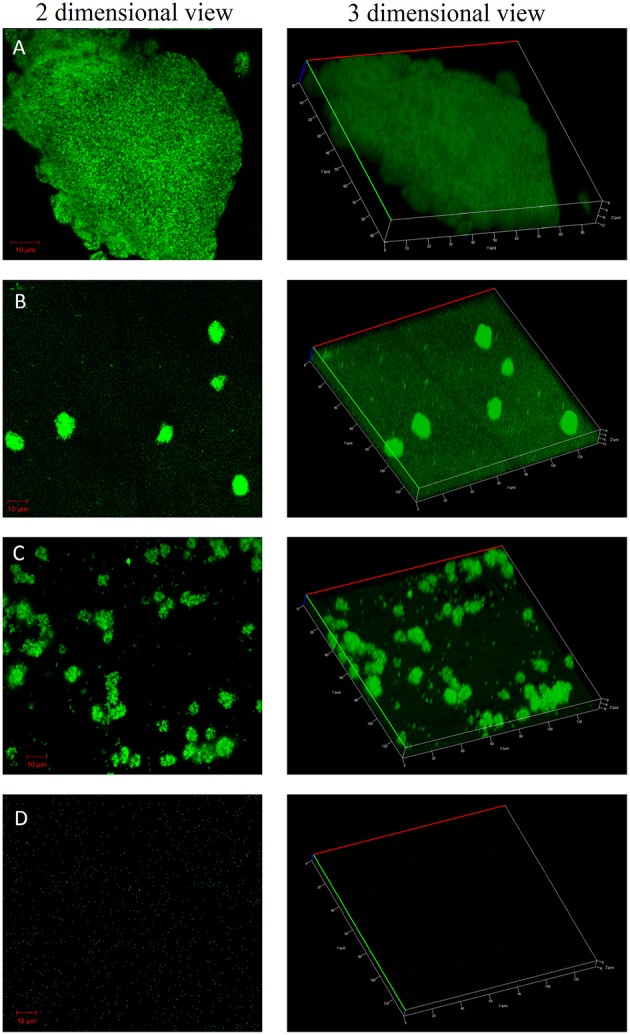
**Effect of ComAI treatments alone as well as in synergy on *S. mutans* 5SM (clinical isolate) biofilms**. Live/Dead staining of bacterial biofilm observed under confocal laser scanning microscopy (green color-Syto 9). **(A)** Control biofilm, **(B)** ComAI treated biofilm (15 μM), **(C)** Fluoride treated biofilm (500 ppm), **(D)** Synergistic activity of ComAI and fluoride (31.25 ppm fluoride and 15 μM ComAI).

The confocal microscopic images of biofilm stained with BacLight Live/Dead stain were shown in Figures 4, 5. Absence of red fluorescence in the merged images from the two filter sets i.e., for live cells (Syto 9, green) and dead cells (Propidium iodide, red) showed that the treatments did not affect the survival of bacteria and the compounds are not antibacterial. This was further confirmed by plating the planktonic cells and growth inhibition was not observed (data not shown). Fifty percent biofilm inhibition was observed in ComAI (1.89 μM) treated biofilms. Similar results were observed in ComAI^1^ (3.75 μM) treated biofilms. No significant biofilm inhibition was observed in biofilms when treated with fluoride alone whereas in case of combinatorial treatments, highly significant biofilm inhibition was observed for both *S. mutans* strains taken in this study.

## Discussion

The exploration for novel therapeutic compounds that act as antibiofilm and anti-virulent compounds such as 7-fluoroindole, 5-fluorouracil (Attila et al., 2009; Lee et al., 2012) rather than antibacterial in nature, is one amongst the major research focus these days to overcome drug resistance. Furthermore, such anti-virulent compounds when used in combination with commercially existing protective agents, results in significant reduction in the over use of these agents. In the present study, disubstitutedurea compounds were screened and synthesized and tested for its antibiofilm potential toward *S. mutans*. In addition, combinatorial treatment with commercially used anti-caries agents such as fluoride and chlorhexidine was evaluated *in vitro* (Somani et al., 2010; Tong et al., 2011).

In light of our present *in silico* studies, we have found that disubstituted ureas have better binding affinity to ComA, a bacteriocin associated ABC transporter. Moreover, all the derived compounds have been shown to bind at the active site of PEP domain of ComA. Blocking of the active site will render the cleaving property of PEP domain inactivated. Hence, ComA inhibitors and its derivatives were chemically synthesized and shown to inhibit the formation of biofilm. Earlier reports by Mustafa et al. have shown anti-cancerous and enzyme inhibitory effects of 1,3-disubstituted ureas (Mustafa et al., 2014). Similarly, fluoride at higher concentrations has been reported to have antibacterial effects but has not been shown to address biofilm inhibition, a major virulence trait of the disease effectively (Zheng et al., 2015). Earlier report by Murata et al. has shown that fluoride at a concentration of 125 ppm act synergistically along with 7-epiclusianone (a naturally occurring compound) to enhance the cariostatic effect of fluoride (Murata et al., 2010). Similarly, biofilm inhibition using calcium fluoride nanoparticles was observed by Kulshrestha et al. (2016).

In the present study, we have reported the effective application of chemically synthesized 1,3-disubstituted ureas that act as antibiofilm agents against standard and clinical isolates but do not have any antibacterial property which is a desirable characteristic as far as multi-drug resistance is concerned. This can be attributed to the fact that, since, these compounds were designed to selectively block the ComA, and thus, the process of cell to cell communication is inhibited leading to expression of virulence factor such as biofilm formation. Moreover, 1,3-disubstituted ureas, when used in combination with fluoride, significantly reduced the concentration of fluoride required for prevention of dental caries (1000 ppm used in the present formulations). In our present study, we have reported the concentration of 31.25 ppm which is found to act synergistically along with ComAI and its derivatives in all the clinical as well as standard isolates which is relatively very less fluoride concentration when compared to the presently used formulations. This suggests that the combinatorial treatment was effective and ensures the usage of antimicrobial products within the limits that may reduce or eliminate the chance of resistance development. However, similar results were not observed with chlorhexidine.

Enhancement of cario-preventive effects of fluoride by integrating the natural or synthesized compounds in oral preparations affecting the virulence of cariogenic bacteria will lead to the better dental care. Fluoride along with 1,3-disubstituted ureas clearly has potential to reduce the incidence of dental caries at a very low concentrations thus, avoiding the harmful effects of increased exposure of fluoride to individuals. Collectively, based on the *in vitro* studies, our data shows that 1,3-disubstituted ureas reduces biofilm formation when used alone or in combination with fluoride without reducing its cariostatic properties. The combination of fluoride with 1,3-disubstituted ureas may provide a potentially useful alternative to the current chemo-based anti-caries strategies to prevent dental caries disease. However, additional studies are essential to elucidate the molecular mechanisms of action of these agents and optimize the combinations. This would enhance the present effectiveness of anti-caries chemotherapy.

## Author contributions

All authors listed, have made substantial, direct and intellectual contribution to the work, and approved it for publication.

### Conflict of interest statement

The authors declare that the research was conducted in the absence of any commercial or financial relationships that could be construed as a potential conflict of interest.

## Footnotes

^1^Clinical and Laboratory Standards Institute (CLSI). CLSI Approved Standard—Tenth Edition). Methods for Dilution Antimicrobial Susceptibility Tests for Bacteria That Grow AerobicallyCLSI document M07-A10 ISBN 1-56238-987-4; ISBN 1-56238-988-2.

## Supplementary material

The supplementary information includes the NMR data (1H and 13C NMR) for all the synthesized compounds.
