# Supplementary material for: Combinatorial Effects of Aromatic 1,3-Disubstituted Ureas and Fluoride on In vitro Inhibition of Streptococcus mutans Biofilm Formation
Source: Front Microbiol. 2016 Jun 6;7:861. doi: 10.3389/fmicb.2016.00861 (PMC4893485; doi:10.3389/fmicb.2016.00861)
Supplement: Supplementary file 1 [file DataSheet1.DOCX]

Supplementary Material

**Combinatorial effects of aromatic 1,3 – disubstituted ureas and fluoride on *in vitro* inhibition of *Streptococcus mutans* biofilm formation**

**Gurmeet Kaur, P. Balamurugan, C. Uma Maheswari, Anitha A. and S. Adline Princy***

***Correspondence -** adlineprinzy@biotech.sastra.edu

**Supplementary Data: ^1^H and ^13^C NMR**

**ComAI**

^1^H NMR (300 MHz, DMSO) δ 8.59 (d, J = 4.9 Hz, 1H), 7.29 (d, J = 5.1 Hz, 1H), 7.17 (dd, J = 19.0, 7.4 Hz, 2H), 6.77 (s, 1H), 2.26 (d, J = 5.0 Hz, 3H).

^13^C NMR (75 MHz, DMSO) δ 152.5, 139.6, 137.9, 128.6, 122.5, 118.7, 115.3, 21.2.

**ComAI^1^**

^1^H NMR (300 MHz, DMSO) δ 8.70 (d, J = 51.5 Hz, 2H), 7.52 – 7.42 (m, 2H), 7.32 (dt, J = 7.7, 4.3 Hz, 3H), 7.24 – 7.12 (m, 2H), 6.80 (d, J = 7.1 Hz, 1H), 2.27 (s, 3H).^13^C NMR (75 MHz, DMSO) δ 152.4, 139.4, 138.7, 137.9, 128.6, 122.7, 119.6, 118.8, 115.5, 21.2.

**ComAI^2^**

^1^H NMR (300 MHz, DMSO) δ 8.49 (s, 1H), 8.45 (s, 1H), 7.36 – 7.26 (m, 3H), 7.15 (dd, J = 16.8, 9.4 Hz, 2H), 6.89 – 6.83 (m, 2H), 6.77 (d, J = 7.2 Hz, 1H), 3.71 (s, 3H), 2.27 (s, 3H).

^13^C NMR (75 MHz, DMSO) δ 154.4, 152.7, 139.8, 137.9, 132.7, 128.5, 122.3, 119.9, 118.6, 115.2, 113.9, 55.1, 21.2.

**ComAI^3^**

^1^H NMR (300 MHz, DMSO) δ 9.52 (s, 1H), 8.17 (d, J = 9.3 Hz, 2H), 7.92 (s, 1H), 7.69 (d, J = 9.3 Hz, 2H), 6.90 (s, 2H), 2.24 (s, 3H), 2.16 (s, 6H). ^13^C NMR (75 MHz, Acetone) δ 153.7, 147.9, 142.5, 137.1, 136.8, 132.9, 129.5, 125.7, 118.4, 20.9, 18.5.

**ComAI^4^**

^1^H NMR (300 MHz, DMSO) δ 8.45 (s, 1H), 8.41 (s, 1H), 7.36 – 7.29 (m, 4H), 7.07 (d, J = 8.3 Hz, 2H), 6.88 – 6.83 (m, 2H), 3.71 (s, 3H), 2.23 (s, 3H).

13C NMR (75 MHz, DMSO) δ 154.4, 152.8, 137.3, 132.8, 130.4, 129.1, 119.9, 118.2, 113.9, 55.1, 20.3.

**ComAI^1’^**

^1^H NMR (300 MHz, DMSO) δ 8.76 (s, 1H), 8.59 (s, 1H), 7.47 (dd, J = 9.4, 2.5Hz, 2H), 7.39 – 7.25 (m, 4H), 7.09 (d, J = 8.3 Hz, 2H), 2.24 (s, 3H).^13^C NMR (75 MHz, DMSO) δ 152.4, 138.8, 136.9, 130.8, 129.1, 128.5, 125.2, 119.6, 118.4, 20.3.
